# Two kinds of rare light chain cast nephropathy caused by multiple myeloma: case reports and literature review

**DOI:** 10.1186/s12882-021-02250-z

**Published:** 2021-01-28

**Authors:** Li-Jun Sun, Hong-Rui Dong, Xiao-Yi Xu, Guo-Qin Wang, Hong Cheng, Yi-Pu Chen

**Affiliations:** Division of Nephrology, Beijing Anzhen Hospital, Capital Medical University, No.2 Anzhen Street, Chaoyang District, Beijing, 100029 People’s Republic of China

**Keywords:** Multiple myeloma, Light chain, Cast nephropathy, Amyloid casts, Crystalline casts, Acute kidney injury, Renal biopsy, Urinalysis

## Abstract

**Background:**

Light chain cast nephropathy (LCCN) is the most common renal disease caused by multiple myeloma (MM). In addition to ordinary light chain protein casts, there are a few rare casts with unique shapes, including light chain amyloid casts (LCAC) and light chain crystal casts (LCCC).

**Case presentations:**

Here, we report two patients. Patient 1 is a 72-year-old man who was clinically diagnosed with MM and acute kidney injury (AKI). Pathological examination of a renal biopsy revealed that there were many amyloid casts in the distal tubules that had a lightly-stained central area and a deeply-stained burr-like edge. The marginal zone of the cast was positive for Congo red staining and contained numerous amyloid fibers, as observed by electron microscopy. No systemic amyloidosis was found. The patient received 4 courses of bortezomib-based chemotherapy, and then, his MM achieved partial remission. Patient 2 is a 57-year-old man who was also clinically diagnosed with MM and AKI. Pathological examination of a renal biopsy showed that there were many crystalline casts in the distal tubules that were fully or partially composed of crystals with different shapes, including rhomboid, needle, triangle, rectangle and other geometric shapes. Congo red staining was negative. Crystals were also detected in the urine of this patient. After 9 courses of treatment with a bortezomib-based regimen, his MM obtained complete remission and his renal function returned to normal.

**Conclusions:**

LCAC and LCCC nephropathy caused by MM are two rare types of LCCN, and both have their own unique morphological manifestations. LCAC nephropathy may not be accompanied by systemic amyloidosis. The diagnosis of these two unique LCCNs must rely on renal biopsy pathology, and the discovery of urine crystals is of great significance for indicating LCCC nephropathy.

**Supplementary Information:**

The online version contains supplementary material available at 10.1186/s12882-021-02250-z.

## Background

Multiple myeloma (MM) is a malignant neoplasm caused by clonal hyperplasia of plasma cells in the bone marrow [1, 2]. Renal impairment is common and is frequently the initial presentation of MM. It is reported that 20 to 50% of patients had kidney involvement at the time of MM diagnosis [1–3]. MM can cause a variety of types of renal damage, among which, light chain cast nephropathy (LCCN) is the most common and usually leads to acute kidney injury (AKI). Moreover, MM can also cause renal amyloidosis, monoclonal immunoglobulin deposition disease, type I cryoglobulinemic glomerulonephritis, and rarely light chain proximal tubulopathy (LCPT) [1–3].

Light chain casts are formed through the following processes: a large number of monoclonal free light chain proteins are synthesized by clonal hyperplasic plasma cells in the bone marrow and secreted into circulation. These proteins then pass through the glomerular filtration membrane to reach the renal tubules and are endocytosed by proximal tubular epithelial cells. When the endocytosis of the epithelial cells reaches saturation, the excess light chain proteins remain in the tubular fluid and flow through Henry’s loop, where they combine with uromodulin and then form casts in the distal tubular lumen [2–4]. The light chain casts usually show a “fractured” appearance and are surrounded by mononuclear cells and multinuclear giant cells. The casts appear red with hematoxylin-eosin (HE) staining, polychromatic with Masson trichrome staining, and light or negative with periodic acid-Schiff (PAS) staining and periodic acid silver methenamine (PASM) staining. Immunofluorescence (IF) or immunohistochemical examination reveals that the light chain protein in casts is monoclonal light chain λ or κ [3, 5, 6]. However, in rare cases of MM, some casts with unusual shapes can also be seen, including light chain amyloid casts (LCAC) and light chain crystal casts (LCCC), can also be seen. In recent years, we have diagnosed one patient with LCAC nephropathy and one patient with LCCC nephropathy. Here, we provide a case report and literature review on these patients.

## Case presentations

### Case 1

A 72-year-old man was admitted to our hospital due to weakness and elevated serum creatinine level. Eight months prior to admission, (+) urinary protein and 115.1 μmol/L serum creatinine (reference value 57–111 μmol/L) were observed in this patient. His serum creatinine level had increased to 282.8 μmol/L 3 months prior to admission and to 461.4 μmol/L 6 days prior to admission. The patient had a 15-year history of hypertension. On admission, the patient’s blood pressure was 132/86 mmHg. Physical examination found no remarkable abnormality except a slightly pale face. The laboratory tests results were as follows: hemoglobin level was 97 g/L; urine protein (dipstick test) was (+); 24-h proteinuria was 4.19 g; serum albumin level was 35.8 g/L; serum globulin level was 20.1 g/L; serum calcium level was 3.39 mmol/L; serum creatinine level varied between 445.9 and 682.7 μmol/L; urine osmotic pressure was 330 mOsm/kg·H_2_O (reference value 600–1000 mOsm/kg·H_2_O); urine α-1 microglobulin level was 190 mg/L; serum IgA, IgG and IgM levels were all decreased (0.33 g/L, 6.16 g/L and 0.06 g/L, respectively); and serum complement C3 and C4 levels were normal. In addition, immunofixation electrophoresis revealed that there were monoclonal IgG κ and κ light chain in the patient’s serum and urine, respectively (Supplementary file 1: Fig. S1). Serum free light chain measurement (N Latex FLC kappa & lambda assay) showed that the κ light chain level was 9270.0 mg/L (reference value 6.7–22.4 mg/L), the λ light chain level was 58.2 mg/L (8.3–27 mg/L) and the κ/λ ratio was 159.3 (0.31–1.56). Bone marrow smear examination showed that the percentage of immature plasma cells was 27.5%. X-ray examination showed multiple osteolytic lesions in the pelvis. Thus, IgG κ-type MM was diagnosed.

**Fig. 1:**
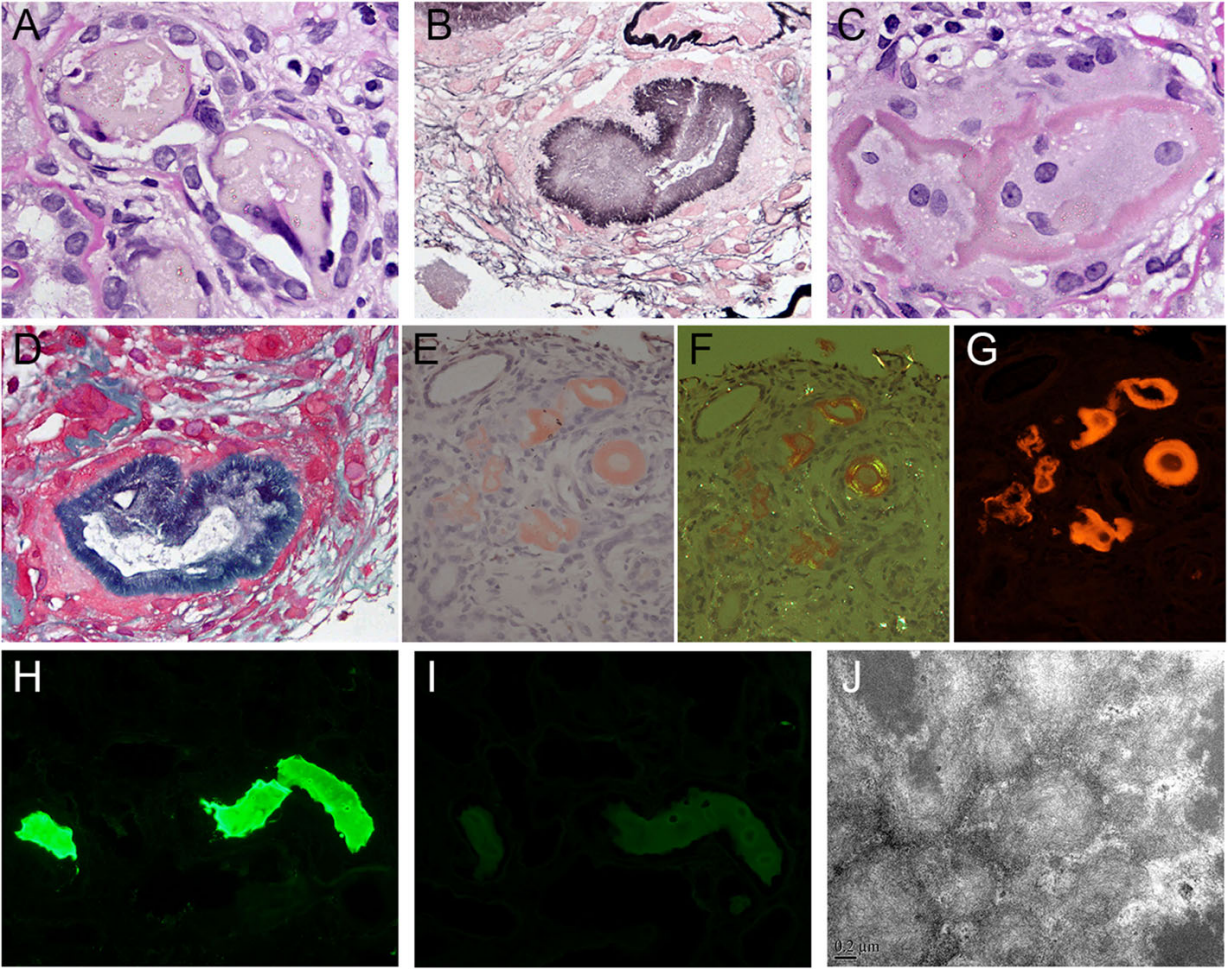
Pathologic findings of kidney biopsy tissue in Case 1. An ordinary protein cast with a “fractured” appearence and peripheral cellular reaction, which was weakly stained in PAS staining (**a**) (original magnification× 1000). A cast with an unique shape, that had a lightly stained central area and a deeply stained burr-like edge in PASM-Masson trichrome double staining (**b**), PAS staining (**c**) and Masson trichrome staining (**d**) (original magnification × 1000). The above unique casts stained with Congo red under ordinary light microscope (**e**), polarized light microscope (**f**) and fluorescence microscope (**g**) (original magnification × 400). The unique casts with strong staining of κ light chain (**h**) and no staining of λ light chain (**i**) (fluorescence micrographs × 400). Numerous randomly arranged unbranched fibrils with a diameter of 8–12 nm in a unique cast (**j**) (electron micrograph × 50,000)

A renal biopsy was performed. Light microscopy revealed that there were 15 glomeruli in the section, of which, 7 were of ischemic sclerosis showing ‘wrinkling collapse’ of the glomerular capillary tuft and the rest were not significantly abnormal. There were many casts in the lumen of distal tubules. Approximately 35% of the casts were ordinary light chain protein casts with a “fractured” appearance surrounded by mononuclear cells and occasionally by multinucleated giant cells (Fig. 1 A). Approximately 65% of the casts showed a unique shape with a lightly-stained central area and a deeply-stained burr-like edge, which was black with PASM staining, blue with Masson trichrome staining and fuchsia with PAS staining (Fig. 1b to d). The marginal zone of the casts was Congo red positive (Fig. 1e to g), while the glomeruli and renal arterioles were negative. There was multifocal interstitial fibrosis (accounting for 65% of the interstitial area) with moderate mononuclear cell infiltration and renal tubule atrophy. The walls of the renal arterioles were moderately thickened with hyaline deposits. Immunofluorescence examination showed κ light chain restriction in the unique casts (κ light chain was strongly positive, while λ light chain negative) (Fig. 1h and i). Electron microscopy revealed that numerous randomly arranged unbranched fibrils with a diameter of 8–12 nm existed in the marginal zone of the unique casts (Fig. 1j). Therefore, the pathological diagnosis was LCAC nephropathy, κ-type, and benign hypertensive nephrosclerosis. In addition, bone marrow biopsy and periumbilical subcutaneous adipose pad biopsy were also performed. The pathological diagnosis of the bone marrow was plasmacytoma. Congo red staining was negative in the bone marrow, subcutaneous adipose tissue and arterioles.

The patient received 3 courses of bortezomib, dexamethasone and cyclophosphamide triple chemotherapy, and then 1 course of bortezomib, dexamethasone, cyclophosphamide and etoposide quadruple chemotherapy. After treatment, the monoclonal band of serum immunofixation electrophoresis disappeared and the ratio of serum free light chain κ/λ decreased to 71.4, but the renal function did not improve. Thereafter, the patient ceased chemotherapy, switched to traditional Chinese medicine, and underwent maintenance hemodialysis.

### Case 2

**Fig. 2:**
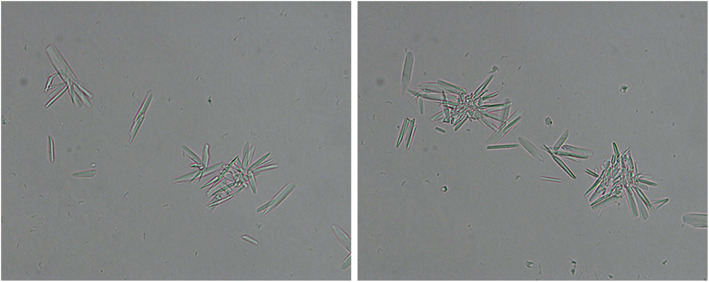
Light microscopy of urinary sediment after centrifugation. Many clustered or scattered crystals of needle shape and other shapes in the urine (original magnification × 1000)

A 57-year-old man was admitted to our hospital because of bone pain and raised serum creatinine. He has felt multiple bone pain for 1 month. Six days prior to admission, a small amount of proteinuria (+) and slightly elevated serum creatinine levels, reaching 162.9 μmol/L, were observed in this patient. His significant medical history was 26 years of mild hypertension. At admission, the patient’s blood pressure was 131/82 mmHg and physical examination showed no abnormalities. His hemoglobin level was 105 g/L. Urine protein (dipstick test) was trace (±) and 24-h proteinuria was 5.4 g. Many clustered or scattered crystals of needle shape and other shapes were detected in his urinary sediment by light microscopy (Fig. 2). The serum albumin level was 39.8 g/L, and the globulin level was 31.0 g/L. The serum calcium level was 3.66 mmol/L. The serum creatinine level was 223.5 μmol/L. The urine osmotic pressure was 250 mOsm/ kg·H_2_O, and the urine α-1 microglobulin level was 33.1 mg/L. The serum IgA, IgG and IgM levels were all decreased (0.37 g/L, IgG 6.66 g/L and IgM 0.06 g/L, respectively). The serum complement C3 and C4 levels were normal. Monoclonal λ light chain was detected in the serum and urine by immunofixation electrophoresis (Supplementary file 1: Fig. S2). The serum free light chain assay showed that the κ light chain level was 15.8 mg/L, the λ light chain level was 1310.0 mg/L and the k/λ ratio was 0.012. Bone marrow smear examination revealed that the percentage of immature plasma cells was 36.5%. Multiple osteolytic lesions in the skull, mandible and pelvis were observed in the X-ray films. Thus, the diagnosis of λ-type light chain MM was established.

**Fig. 3:**
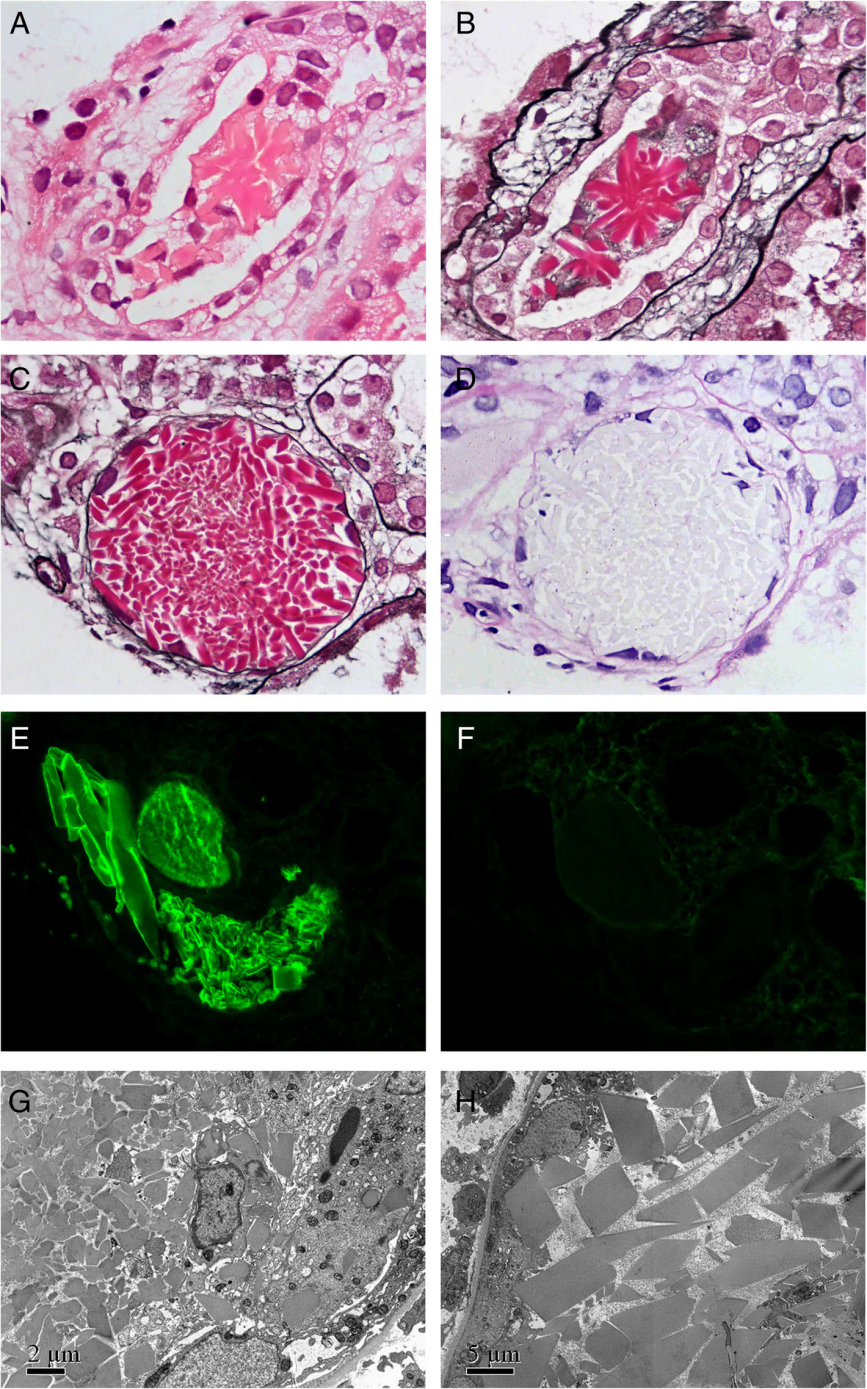
Pathologic findings of kidney biopsy tissue in Case 2. A cast containing crystals of various geometric shapes, which was partially surrounded by mononuclear cells (**a** and **b**) (HE staining and PASM-Masson trichrome double staining respectively, both × 1000). A cast consisting of a lot of crystals, around which the tubular epithelial cells have been destroyed or lost (**c** and **d**) (PASM-Masson trichrome double staining and PAS staining respectively, both × 1000). Casts with strong staining of λ light chain (**e**) and no staining of κ light chain (**f**) (fluorescence micrographs × 400). A lot of crystals with different sizes and shapes in a cast (**g** and **h**) (electron micrographs × 8000 and × 4000, respectively)

A renal biopsy was carried out. Light microscopy revealed there were 1 glomeruli in the renal biopsy tissue sections and they were all basically normal. In the distal tubular lumen, there were many casts, of which, approximately 65% were ordinary light chain protein casts that usually had a “fractured” appearance and were surrounded by cellular reaction. The rest, 35%, were unique crystalline casts, which were fully or partially composed of crystals with different shapes, including rhomboid, needle, triangle, rectangle and other geometric shapes. These casts were red with HE staining and Masson trichrome staining, but not colored with PAS staining and PASM staining. Cellular reactions surrounding some crystalline casts could also be seen (Fig. 3a to d). The renal interstitium showed focal fibrosis (less than 25% of the total interstitial area) with mild mononuclear cell infiltration and renal tubule atrophy. No crystal deposition was found in the renal arteriolar lumen. Congo red staining in the renal parenchyma was negative. Immunofluorescence examination showed that the λ light chain staining of the crystalline casts was positive, while the κ light chain was negative, suggesting λ light chain restriction (Fig. 3e and f). Electron microscopy revealed many rhombic, rectangular, triangular or irregular crystals in the unique casts (Fig. 3g and h). Hence, the pathological diagnosis was LCCC nephropathy λ-type. Bone marrow biopsy was also performed, and its pathological diagnosis was plasmacytoma. No crystal could be found in the bone marrow tissue.

After diagnosis was confirmed the patient received bortezomib, lenalidomide and dexamethasone triple chemotherapy for a total of 9 courses. His MM achieved complete remission. Serum and urine immunofixation electrophoresis, serum free light chain concentration and serum IgA, IgG, IgM levels had all returned to normal, and the serum creatinine level and estimated glomerular filtration rate also returned to normal.

## Discussion and conclusions

LCCN is the most common MM-related renal disease. According to a Mayo Clinic report, LCCN accounts for 33% of all cases of MM-related kidney diseases [1]. However, LCAC nephropathy and LCCC nephropathy are very rare variants. We used Medline and EMBASE database retrieval and manual retrieval to collect articles and case reports of LCAC and LCCC nephropathy written in English for literature review. Abstracts of conference proceedings that were not published in full were not included (Supplementary file 2).

In 1962, when Vassar et al. [7] and Azzopardi et al. [8] first reported LCACs when they observed these unique morphological casts in autopsies of patients with MM. From 1962 to 1980, LCACs were found in approximately 55 autopsy patients with MM, and in about 2/3 of these patients, amyloid casts were diagnosed only by methyl violet staining and/or thiocyanin T staining [7–12]. From 1980 to 2020, only 25 patients with LCACs caused by MM were identified (renal biopsy in 24 cases and autopsy in 1 case), and the diagnosis of amyloid casts in these patients was based on Congo red staining and/or electron microscopy [6, 13–21] (Table 1). In addition, in three other papers the authors mentioned that they also observed LCACs in some patients with renal damage caused by MM, but they did not provide a detailed description [22–24].

**Table 1:** Light chain amyloid in tubular casts and other tissues in multiple myeloma

References	Year	No of cases	LCAC			Other kidney tissue amyloidosis	Extrarenal tissue amyloidosis
			Location	Pattern	Type		
Vassar PS [7]	1962	33^a^	ND	Rimmed Laminated	ND	PTC cytoplasm in several cases	ND
Azzopardi JG [8]	1962	1^a^	Distal	Laminated	ND	No	No
Azzopardi JG [9]	1966	1^a^	ND	Rimmed Laminated	ND	No	Yes in skin and Pericardium, etc.
Friman C [10]	1970	1^a^	Distal	Laminated	ND	No	Yes in tumour, No in other organs
Limas C [11]	1973	15^a^	convoluted tubules, loops of Henle, etc.	Rimmed	ND	PTC cytoplasm in 3 cases	No ^c^
Melato M [12]	1980	4^a^	ND	Rimmed Laminated Homogeneous	ND	No	No
El-Zoghby Z [13]	2007	1^b^	ND	Rimmed	λ	PTC cytoplasm	Yes in BM and joint
Nasr SH [14]	2008	1^b^	ND	Rimmed	λ	No	ND
Sethi S [15]	2009	1^b^	ND	Rimmed	λ	No	No in BM
Sharma A [16]	2014	1^b^	ND	Rimmed	λ	No	No in BM
Kato H [6]	2015	1^b^	Distal	Rimmed Laminated Homogeneous	λ	No	ND
Iliuta IA [17]	2016	1^b^	Many segments	Homogeneous	λ	PTC cytoplasm	ND
Kurien AA [18]	2018	1^b^	ND	Rimmed Homogeneous	λ	PTC cytoplasm	ND
Gibier JB [19]	2018	16^b^	ND	Rimmed Laminated Homogeneous	λ^△^ in 13 cases	PTC cytoplasm in 4 cases	Yes in 5 cases
Rajagopal MD [20]	2018	1^b^	Distal	Laminated	λ	No	No in BM and salivary gland
Ichimata S [21]	2020	1^a^	ND	Laminated	λ	PTC cytoplasm	Yes in lungs and heart
Our case		1^b^	Distal	Rimmed	κ	No	No in BM and arterioles

*BM* bone marrow, *LCAC* light chain amyloid casts, *MM* multiple myeloma, *ND* not detailed, *PTC* proximal tubular cell

^a^ Autopsy kidney tissues; ^b^ Kidney biopsy tissues

^c^ Amyloid deposition was also found in the extrarenal organs of 8 elderly patients with amyloid casts. However, their amyloid deposition was not different from that of the age-matched controls, so it may not be related to the amyloid casts

Unlike the ordinary light chain protein cast, LCAC has a unique shape. In most cases, it has a lightly stained central area and a deeplystained burr-like edge, which is black, blue and fuchsia with PASM, Masson trichrome and PAS staining, respectively. Positive Congo red staining and electron microscopy can confirm its amyloid properties [15, 16, 18, 19]. Under polarized light microscopy, ordinary light microscopy and fluorescence microscopy, Congo red staining exhibits apple green double refraction, brick red and bright red, respectively [19]. In addition to the abovementioned typical pattern, the amyloid deposits also have other distribution forms in the cast, such as lamination form, which can present as two or more layers, and sometimes in a tree-ring shape [6–10, 12, 19–21], and homogeneous form, which is composed of clumped homogeneous deposits distributed in the whole cast [6, 12, 17, 19]. Electron microscopy shows numerous randomly arranged unbranched fibrils with a diameter of 8–12 nm in the amyloid structures of the casts [6, 13, 15–17, 19, 21]. Immunofluorescence or immunohistochemical examination reveals that the light chain in the cast has monoclonal properties, that is, only λ or κ light chain is present [6, 13–16, 18–21]. The morphological characteristics of the casts in Case 1 of this paper are completely consistent with those of LCAC above. Furthermore, in LCAC nephropathy, the amyloid casts usually coexist with the ordinary light chain protein casts. Gibier et al. [19] reported 17 cases of LCAC nephropathy, of which 16 cases were caused by MM. Among these cases, the proportion of amyloid casts in the total casts was < 5% in 9 cases, 5–25% in 3 cases and > 25% in 5 cases. In Case 1 of this paper, the amyloid casts accounted for 65% of all the casts.

The mechanism of LCAC formation remains unclear. It is known that the free light chains with a low molecular weight (approximately 20–25 kDa) can pass through the glomerular filtration membrane, while the amyloid fibrils with larger size cannot. Therefore, it can be inferred that the LCACs are formed in the tubules [15, 17, 18]. There are two hypotheses. One possible mechanism is that the light chain proteins filtered into Bowman’s space or the renal tubular lumen are affected by some environmental factors (such as the pH value of the filtrate and high concentration urea) to change their conformation and become amyloid proteins with β-fibril structure, and then aggregate to form LCACs in the distal tubules [6, 15, 17, 19, 20]. Another explanation is that the filtered light chain proteins are endocytosed by proximal tubular epithelial cells, undergo a conformational change under the action of lysosomal enzymes and obtain the properties of amyloid; these altered proteins are then discharged from the cells into the lumen to form the LCAC in the distal tubules [11, 15, 18–20]. The later hypothesis is supported by the fact that LCACs often coexist with amyloid light chain-mediated proximal tubulopathy [11, 13, 17–19].

Many patients with LCAC nephropathy do not have amyloid deposits in the glomeruli, tubular epithelial cells, renal small arteries and renal interstitium [6, 8–10, 12, 14–16, 20], nor amyloidosis of extrarenal organs [8, 11, 12, 15, 16, 20]. Even in some autopsy cases, no amyloid lesions in the above sites can be found [8, 12]. The same is true for the patient described in Case 1 of this paper, whose renal parenchyma and bone marrow were negative for Congo red staining. Therefore, a question is raised; is there a link between LCAC and systemic amyloidosis? In 2018, a retrospective large sample study published by Gibier et al. [19] brought the answer to this question to light. After careful and systematic examinations of the tissues of the extrarenal organs (including biopsy tissues and surgical specimens) of patients with LCAC nephropathy caused by MM, the authors did observe amyloid deposits in some extrarenal organs and found that the formation of intratubular LCACs occurred earlier than extrarenal organ amyloidosis. Thus, that study suggests that LCAC may be a precursor of systemic amyloidosis. Based on the findings by Gibier et al. [19], we believe that all patients with LCAC should be carefully examined for the existence of systemic amyloidosis, and if not, a long term follow-up should be performed.

In the literature, only Gibier et al. [19] implemented a controlled cohort study of small samples that compared the response to treatment between the LCCN patients with and without amyloid casts. After treatment with bortezomib and immunomodulatory drug (lenalidomide or thalidomide)-based regimens, the hematological response (at least partial response) and renal response (defined by estimated glomerular filtration rate ≥ 30 ml/min/1.73 m^2^ and/or independence from dialysis at 3 months) were achieved in 68 and 32% of patients, respectively. The hematological and renal responses were not significantly different between the two groups. In addition, there were 3 case reports that described the renal response of patients with LCAC nephropathy after treatment. None of these patients exhibited significant improvement of renal function [13, 18, 20]. In Case 1 of this paper, MM achieved partial remission after 4 courses of chemotherapy, but renal function did not improve concomitantly. The poor renal response of this patient may be caused by two factors: the response to therapy of LCAC nephropathy itself is not good, and the AKI of this patient occurred on the basis of chronic kidney disease (benign hypertensive nephrosclerosis with chronic renal insufficiency). Taken together, the response to therapy of LCAC nephropathy need to be further observed by expanding the number of cases.

Regarding LCCC caused by MM, the earliest data we retrieved were two individual case reports published by Silk [25] and Neumann [26] in 1949, respectively. However, it is said that LCCC was first reported by Löhlein in 1921 and published in a journal of pathology written in German [25]. In the published English literature, we retrieved 12 cases of LCCC confirmed by autopsy from 1949 to 1989 (one of the patients underwent renal biopsy before death) [25–30] and 27 cases of LCCC diagnosed by renal biopsy from 1987 to 2020 [31–43] (Table 2). Moreover, in two other papers, the authors mentioned that they also observed LCCC, but they did not provide detailed descriptions [44, 45].

**Table 2:** Light chain crystals in tubular casts and other tissues in multiple myeloma

References	Year	No of cases	LCCC		Other kidney tissues involved	Extrarenal tissue involved
			Location	type		
Sikl H [25]	1949	1^a^	Distal	ND	PTC cytoplasm	NPC in medulla of the kidney
Neumann V [26]	1949	1^a^	All segments	ND	Bowman’s capsule Interstitial cells	Bone marrow, NPC in tumour area
Schubert GE [27]	1972	7^a^	Distal	ND	PTC cytoplasm Interstitium	Bone marrow
Chejfec G [28]	1983	1^ba^	ND	λ	No	Lung
Dornan TL [29]	1985	1^a^	ND	λ	Bowman’s capsule Blood vessel	Interstitial tissue of the heart
Truong LD [30]	1989	1^a^	Distal	κ	PTC cytoplasm Glomerulus NPC in interstitium	No
Pirani CL [31]	1987	14^bc^	Distal	κ or λ	PTC or DTC cytoplasm (5 cases)	ND
Kanno Y [32]	2001	1^b^	ND	λ?	Bowman’s capsule	Bone marrow
Dehmel B [33]	2003	1^b^	ND	λ	PTC cytoplasm	ND
Chen KJ [34]	2005	1^b^	ND	λ	Glomerulus, Blood vessel, Interstitium	Bone marrow
Toly-Ndour C [35]	2011	1^b^	ND	λ	PTC cytoplasm Bowman’s capsule	Bone marrow
Haider M [36]	2014	1^b^	Distal	λ	No	ND
Luciano RL [37]	2014	1^b^	ND	λ	No	ND
		1^b^	Distal and Proximal	κ	PTC cytoplasm	ND
Gallan AJ [38]	2016	1^b^	ND	λ	No	ND
Kumakura S [39]	2016	1^b^	Proximal	λ	No	ND
Lerner G [40]	2020	1^b^	Distal	κ	PTC cytoplasm	ND
Chou A [41]	2020	1^b^	Distal	κ	Glomerulus, Interlobular arteries	ND
Matsumura H [42]	2020	1^b^	Distal	λ	No	No in bone marrow
Lin Z-S [43]	2020	1^b^	Distal	λ	No	ND
Our case		1^b^	Distal	λ	No	No in bone marrow

*LCCC* light chain crystal casts, *ND* not detailed, *NPC* neoplastic plasma cells, *PTC* proximal tubular cell, *DTC* distal tubular cell

^a^ Autopsy kidney tissues. ^b^ Kidney biopsy tissue

^c^In this paper, definite crystals were detected histologically in 14 cases and features suggestive of crystals in another 4 cases. The 4 suspected cases are not included in this table

The crystals in LCCC vary in size and shape. These crystals can appear as needle-shaped, bar-shaped, spindle-shaped, diamond-shaped, triangle, rectangle, pentagon, hexagon and other geometric shapes [25–43]. Some LCCCs are also surrounded by cellular reactions [25, 26, 28, 31, 32, 37]. LCCCs are usually formed in the distal tubules, but occasionally can also form in the proximal tubules [26, 37, 39]. The staining properties of the crystalline casts are the same as those of ordinary light chain protein casts, appearing eosinophilic by HE staining, polychromatic (mixed red and blue) or fuschinophilic by Masson trichrome staining, pale by PAS staining and lack argyrophilic by PASM staining [38, 39, 42, 46, 47]. If toluidine blue is used to stain semi-thin sections, the crystals appear blue with the best recognition effect [31]. Electron microscopy is also important for identifying and further confirming the crystalline casts. In addition, it has been reported in 3 articles that different shapes of crystals or/and crystalline casts were observed in the urinary sediment of patients with LCCC nephropathy [33, 37, 40]. Luciano et al. [37] believe that urinary sediment microscopy should be performed in all patients with monoclonal light chain-related nephropathy caused by MM, and this approach may provide important clues for the discovery of LCCC nephropathy. The histopathological features of the patient described in Case 2 in this paper are consistent with the LCCC nephropathy described above, and crystals were also found in the urine of this patient.

The mechanism of LCCC formation in patients with MM is still not well understood. There are several hypotheses. First, some light chain proteins more easily form crystals due to their individual characteristics (such as isoelectric point, glycosylation and amino acid sequence). When they are filtered from the glomerulus to the tubular lumen and reach a higher concentration in the tubule fluid, crystals may form under the action of certain local factors (such as a decreased pH value and a slower flow rate of the tubule fluid) [31, 34, 35]. Second, after the filtered light chain proteins are reabsorbed by proximal tubular epithelial cells, if the amount of lysosomal enzymes in the cytoplasm is insufficient, the function of lysosomal enzymes is deficient, or some light chain proteins (such as the κ light chain belonging to the VκI subclass) are resistant to lysosomal enzymes, these light chains will accumulate in the lysosomes, and undergo homogenous polymerization to form crystals [3, 31, 44, 48]. Then, these crystals fall off of the apical surface of the damaged epithelial cells into the tubular lumen, forming LCCCs [45]. Third, LCCC formation may be related with crystalglobulinemia. In this case, monoclonal globulins, or occasionally monoclonal light chains in the systemic vasculature can spontaneously form microcrystals and result in multiple organ embolism [41, 47, 49]. If the microcrystals embolize the glomerular capillaries and cause their destruction, the crystals in the circulation enter Bowman’s space and the tubular lumen and then form crystalline casts in the distal tubules. In the literatures, LCCCs can appear in three states: LCCCs existing alone [36–39, 42, 43], LCCCs coexisting with light chain crystal deposits of the proximal tubular epithelial cells [25, 27, 31, 33, 40], and in a few cases, LCCCs coexisting with light chain crystal deposits of the renal small vessels, glomerular capillaries, Bowman’s space and even extrarenal tissues [26, 29, 30, 32, 34, 35, 41]. These three states may provide some circumstantial evidence for the above three LCCC formation mechanisms. Case 2 in our paper should belong to the first state.

LCCN usually causes AKI clinically, and severe cases often require dialysis treatment. LCCC nephropathy is no exception. Is there any difference in the response to treatment between LCCC nephropathy and ordinary LCCN? To date, too few cases of LCCC nephropathy have been treated, so it is impossible to draw a conclusion. In the literature, Haider et al. [36] and Kumakura et al. [39] each reported one patient with LCCC nephropathy; both patients developed AKI, and one patient underwent hemodialysis. After treatment with bortezomib-dexamethasone regimen, the MM of the patients achieved complete remission or good partial remission, respectively, the elevated serum creatinine levels returned to normal and the hemodialysis was stopped. Chou et al. [41] reported another patient with LCCC nephropathy with AKI undergoing dialysis. After receiving plasma-pheresis and 11 courses of the combined therapy of bortezomib, dexamethasone and cyclophosphamide, her MM achieved complete remission, renal function returned to nearly normal and hemodialysis was no longer needed. The patient described in Case 2 in this paper also obtained the same effects as above after treatment. Therefore, we believe that patients with LCCC nephropathy should not cease treatment for MM, even if they have received dialysis. The prognosis of treated MM and secondary AKI in these patients may still be very good and may be similar to those of ordinary LCCN. Of course, this view needs to be verified by a large number of treatment cases in the future.

In summary, LCAC nephropathy and LCCC nephropathy caused by MM are two rare types of LCCN, and both have their own unique morphological characteristics. LCAC nephropathy may or may not be accompany with systemic light chain amyloidosis. LCCC nephropathy can exist alone or can coexist with crystalline LCPT or crystalglobu-linemia. In the urine of some patients with LCCC nephropathy, crystals may also be detected. The mechanisms of LCAC and LCCC formation are unclear. Is the response to treatment of LCAC nephropathy without systemic light chain amyloidosis better than that of treating LCAC nephropathy with systemic light chain amyloidosis? Is the response to treatment of LCCC nephropathy similar to that of ordinary LCCN? Both questions require further studies.

## Supplementary Information

**Additional file 1.**

## Data Availability

The datasets used and/or analysed during the current study are available from the corresponding author on reasonable request.

## Abbreviations

AKI: acute kidney injury
HE: hematoxylin-eosin
LCAC: light chain amyloid cast
LCCC: light chain crystal cast
LCCN: light chain cast nephropathy
LCPT: light chain proximal tubulopathy
MM: multiple myeloma
PAS: periodic acid-Schiff
PASM: periodic acid silver methenamine
