# Supplementary material for: Two kinds of rare light chain cast nephropathy caused by multiple myeloma: case reports and literature review
Source: BMC Nephrol. 2021 Jan 28;22:42. doi: 10.1186/s12882-021-02250-z (PMC7845023; doi:10.1186/s12882-021-02250-z)
Supplement: Supplementary file 1 — Additional file 1. [file 12882_2021_2250_MOESM1_ESM.docx]

**Supplementary File 1.**


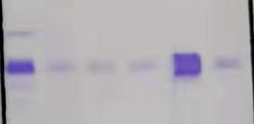

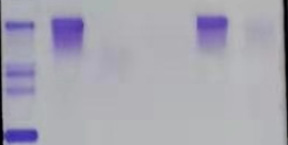


**SP IgG IgA IgM κ λ**

**SP IgG IgA IgM κ λ**

**Figure S1**. Immunofixation electrophoresis. Monoclonal IgGκ in serum (Left) andκlight chain in urine (Right).

**SP IgG IgA IgM κ λ**

**SP IgG IgA IgM κ λ**


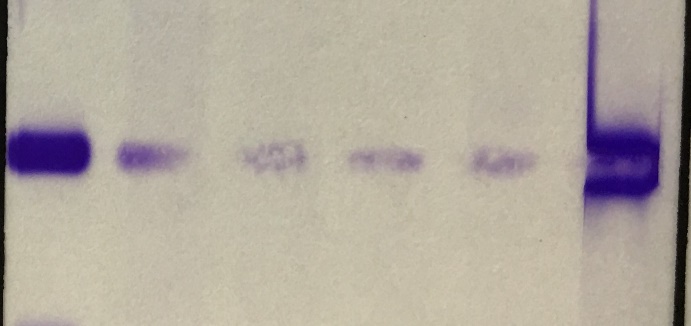


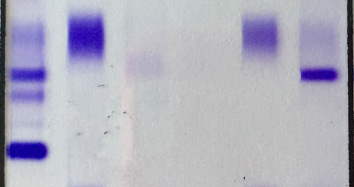


**Figure S2**. Immunofixation electrophoresis. Monoclonal λ light chain in serum (Left) and urine (Right).

**Supplementary File 2.**

**1、Search strategy for MEDLINE (PubMed) database**

**Light chain amyloid cast nephropathy**

| **Step** | **Search Term** |
| --- | --- |
| 1 | Amyloid casts |
| 2 | Intratubular amyloid |
| 3 | Amyloid cast tubulopathy |
| 4 | Unusual casts in multiple myeloma |
| 5 | 1 OR 2 OR 3 OR4 |

Search results: 67

**Light chain crystal casts nephropathy**

| **Step** | **Search Term** |
| --- | --- |
| 1 | Light chain crystal casts |
| 2 | Crystalline cast nephropathy |
| 3 | Crystal cast in multiple myeloma |
| 4 | Crystalline light chain proximal tubulopathy |
| 5 | 1 OR 2 OR 3 OR4 |

Search results: 70

**2、Search strategy for EMBASE database**

**Light chain amyloid cast nephropathy**

| **Step** | **Search Term** |
| --- | --- |
| 1 | Cast nephropathy |
| 2 | Myeloma kidney |
| 3 | Light chain nephropathy |
| 4 | Paraprotein-related kidney disease |
| 5 | Tubular injury |
| 6 | Tubular obstruction |
| 7 | 1 OR 2 OR 3 OR 4 OR5 OR 6 |
| 8 | Exp amyloidosis |
| 9 | Exp amyloid |
| 10 | 8 OR 9 |
| 11 | 7 AND 10 |

Search results: 305

**Light chain crystal casts nephropathy**

| **Step** | **Search Term** |
| --- | --- |
| 1 | Cast nephropathy |
| 2 | Myeloma kidney |
| 3 | Light chain nephropathy |
| 4 | Paraprotein-related kidney disease |
| 5 | Tubular injury |
| 6 | Tubular obstruction |
| 7 | 1 OR 2 OR 3 OR 4 OR5 OR 6 |
| 8 | Crystalline |
| 9 | Crystal |
| 10 | 8 OR 9 |
| 11 | 7 AND 10 |

Search results: 130
